# Quaternary Ammonium Compound Disinfectants Reduce Lupus-Associated Splenomegaly by Targeting Neutrophil Migration and T-Cell Fate

**DOI:** 10.3389/fimmu.2020.575179

**Published:** 2020-10-21

**Authors:** Leila Abdelhamid, Xavier Cabana-Puig, Qinghui Mu, Maryam Moarefian, Brianna Swartwout, Kristin Eden, Prerna Das, Ryan P. Seguin, Libin Xu, Sarah Lowen, Mital Lavani, Terry C. Hrubec, Caroline N. Jones, Xin M. Luo

**Affiliations:** ^1^Department of Biomedical Sciences and Pathobiology, Virginia-Maryland College of Veterinary Medicine, Virginia Tech, Blacksburg, VA, United States; ^2^School of Medicine, Stanford University, Stanford, CA, United States; ^3^Department of Mechanical Engineering, Virginia Tech, Blacksburg, VA, United States; ^4^Translational Biology, Medicine and Health Graduate Program, Virginia Tech, Roanoke, VA, United States; ^5^Department of Medicinal Chemistry, School of Pharmacy, University of Washington, Seattle, WA, United States; ^6^Department of Anatomical Sciences, Edward Via College of Osteopathic Medicine-Virginia Campus, Blacksburg, VA, United States; ^7^Department of Biological Sciences, Virginia Tech, Blacksburg, VA, United States; ^8^Department of Bioengineering, University of Texas, Dallas, TX, United States

**Keywords:** lupus (SLE), neutrophil, quaternary ammonium compound (QAC), autoimmunity, splenomegaly, T cells

## Abstract

Hypersensitivity reactions and immune dysregulation have been reported with the use of quaternary ammonium compound disinfectants (QACs). We hypothesized that QAC exposure would exacerbate autoimmunity associated with systemic lupus erythematosus (lupus). Surprisingly, however, we found that compared to QAC-free mice, ambient exposure of lupus-prone mice to QACs led to smaller spleens with no change in circulating autoantibodies or the severity of glomerulonephritis. This suggests that QACs may have immunosuppressive effects on lupus. Using a microfluidic device, we showed that ambient exposure to QACs reduced directional migration of bone marrow-derived neutrophils toward an inflammatory chemoattractant *ex vivo*. Consistent with this, we found decreased infiltration of neutrophils into the spleen. While bone marrow-derived neutrophils appeared to exhibit a pro-inflammatory profile, upregulated expression of PD-L1 was observed on neutrophils that infiltrated the spleen, which in turn interacted with PD-1 on T cells and modulated their fate. Specifically, QAC exposure hindered activation of splenic T cells and increased apoptosis of effector T-cell populations. Collectively, these results suggest that ambient QAC exposure decreases lupus-associated splenomegaly likely through neutrophil-mediated toning of T-cell activation and/or apoptosis. However, our findings also indicate that even ambient exposure could alter immune cell phenotypes, functions, and their fate. Further investigations on how QACs affect immunity under steady-state conditions are warranted.

## Introduction

Systemic lupus erythematosus (SLE or lupus) is a complex multisystem autoimmune disorder with diverse clinical manifestations (1–3), with enlargement of lymphoid organs as one of the first symptoms (4–6). Genetic mutations can initiate the progression of SLE in susceptible individuals (7–10). However, lupus manifestations are very heterogenic and can vary from one individual to another (11, 12). Aside from genetic factors, this heterogeneity is strongly influenced by environmental factors (13, 14) that could drive the progression of SLE in susceptible individuals and shape treatment outcomes. In this study, we sought to better understand how environmental factors influence the complex pathogenesis of SLE.

Quaternary ammonium compounds (QACs) are ubiquitous biocides (15), antiseptics (16), and widely used surface disinfectants (16). Exposure to QACs is associated with development of topical hypersensitivity (17) and asthma (18) especially in hospital settings (19). Although the exact mechanisms of how QACs modulate immune cell responses are unclear, QAC-induced allergic responses have been attributed to abrogated type 2 innate lymphoid cell responses (20). To understand the possible effects of QACs on innate immunity, we investigated how QACs affected murine macrophages *in vitro*. Exposure to QACs increased the production of pro-inflammatory cytokines but paradoxically reduced the phagocytic potential of murine macrophages (21). Interestingly, effective macrophage responses are required for clearance of autoantigens; and impaired macrophages responses could contribute to the development of autoimmune conditions (22). These findings raised the question of whether QAC exposure would cause immune dysregulation and affect systemic autoimmunity such as lupus. We hypothesized that QACs would exacerbate lupus.

Here, we delineate the effects of ambient exposure to QAC disinfectants on the progression of lupus-like disease in the MRL/lpr mouse model. We show that ambient QAC exposure mitigates lupus-associated splenomegaly, and propose abnormal neutrophil migration and T-cell dysregulation as potential mechanisms behind this surprising finding.

## Materials and Methods

### Animal Housing

MRL/lpr mice were purchased from the Jackson Laboratory (Bar Harbor, ME) and maintained in a specific pathogen-free environment in an AAALAC accredited animal facilities at Virginia Tech. All procedures were carried out according to an approved IACUC protocol 17-128. Due to the challenge of eliminating QACs once contaminated, QAC-free and exposed mice were housed in separate vivaria (23, 24). In addition, QAC-free mice were housed in disposable cages to avoid QAC contamination during cage wash, whereas mice with ambient exposure to QAC were housed in reusable polycarbonate cages. With the exception of disinfectants, all other factors, including diet (hormone-free NIH-31 Modified 6% Mouse/Rat Diet), bedding (shredded paper), and light cycle (12-h light/dark), were consistent among the facilities. Food and water were provided *ad libitum*. QAC-based disinfectants CP-64 or Labsan 256 were used under QAC-exposed conditions, while ethanol was used in the QAC-free environment. To generate QAC-free mice, MRL/lpr mice were bred for at least 2 generations in the QAC-free facility (IDU) to eliminate any contamination from vendor sources (23, 25). QAC-exposed mice were exposed either *via* cage wash only (Phase IV, uncontrolled environment) or through the use of a QAC-based disinfectant inside the room (ILSB). Only female mice were analyzed as lupus has a strong sex bias (26–28).

### Measurement of QAC Levels

Multiple cages were sequentially washed with 50 ml LC/MS-grade methanol. QAC residues from the samples were then extracted using Folch solution (chloroform/methanol = 2/1). After extraction, the samples were re-constituted in the LC solvent and then analyzed by UPLC-MS/MS (29). QAC-equivalent units per mouse cage, which were variable due to the nature of ambient exposure, were calculated and presented in **Table S1**.

### Isolation of Bone Marrow (BM)-Derived Neutrophils

BM isolation was performed as previously described (30, 31) with minor modifications. The procedures were performed at room temperature instead of 4°C, as the latter might interfere with neutrophil migration and chemotaxis assays. In addition, we proceeded to enrich neutrophils without red blood cell lysis due to the same concern. A Mouse Neutrophil Enrichment Kit (StemCell Technologies, Vancouver, BC) was used following the manufacturer’s procedures.

### Neutrophil Chemotaxis Assay

A Microfluidic Competitive Chemotaxis-Chip (μC3) device (**Figure S1**) was modified from our previous design (32). Device fabrication was achieved with 50 μl fibronectin (Sigma-Aldrich, St. Louis, MO) at a concentration of 10 μg/ml following our previously reported procedures (32) to recapitulate the extracellular matrix needed for neutrophil adhesion to the device surface (32, 33). The chemoattractant, leukotriene B_4_ (LTB_4_; Cayman Chemical, Ann Arbor, MI), diluted in RPMI supplemented with 10% fetal bovine serum, was loaded into the chemoattractant reservoir at a clinically relevant concentration of 100 nM (32, 34). Time-lapse imaging experiments were performed as previously reported (32). Time-lapse image capture was performed with a NIS-elements (Nikon Inc., Melville, NY). Images were recorded using fluorescent and bright-field channels at 3-min intervals for 8 h. Image analysis was performed with ImageJ.

### Isolation of Splenocytes and *In Vitro* Stimulation

Spleens were harvested aseptically and total splenocytes were isolated as we previously reported (30). To determine T cell proliferation and/or apoptosis potential, splenocytes were stimulated with anti-CD3/anti-CD28 mouse monoclonal antibodies (eBioscience) following the manufacturer’s protocol with minor modifications. For proliferation analysis, splenocytes were initially stained with CellTrace™ CFSE Cell Proliferation Dye (Thermo Scientific). Briefly, 96-well plates were pre-coated overnight with 5 μg/ml anti-CD3e at 4°C, then washed to remove unbound antibodies. CFSE-treated or untreated cell suspension were added at a cell density of 2 × 10^6^ cells/ml in 100 μl/well, and a soluble anti-CD28 antibody was added at a concentration of 1 μg/ml. Cells were incubated at 37°C for 2 to 3 days then analyzed for proliferation or apoptosis using flow cytometry.

### Flow Cytometry

Cells were initially blocked with anti-mouse CD16/32 (eBioscience) then stained with fluorochrome-conjugated antibodies following our previous procedures (30). Briefly, after blocking of Fc receptors, cells were stained with fluorochrome-conjugated antibodies on ice in the dark for 15 min. Exclusion of dead cells was achieved with Zombie Aqua (BioLegend) staining. For quantification of T cells in total splenocytes, the following anti-mouse antibodies were used: CD3-APC, CD4-PE-Cy7, CD8-PerCP-Cy5.5, CD44-PE, CD62L-APC-Cy7, and CD69-Pacific blue (BioLegend). The same panel of antibodies were used for *in-vitro* activated splenocytes. For analysis of apoptosis, FITC-Annexin V Apoptosis Detection Kit (BioLegend) was used together with propidium iodide according to the manufacturer’s procedures. Splenic Gr-1+ cells in the CD11b+CD11c− myeloid population were analyzed using the following anti-mouse antibodies: CD11c-APC, CD11b-PerCP-Cy5, PD-L1-PE-Cy7 (BioLegend), and Gr-1-V540 (BD Bioscience). Analysis was performed with a BD FACSAria II flow cytometer (BD Biosciences). Flow cytometry data were analyzed with FlowJo.

### Immunohistochemistry (IHC)

Splenic tissue embedding, cryosectioning and storage were performed as previously reported (30). Neutrophils were visualized with anti-mouse Ly6G-PE (Biolegend) and IHC was performed as previously described (30, 35). To visualize the interaction between neutrophils and T cells through PD-L1:PD-1 interaction, we used anti-mouse PD-L1-AF405 (R&D Systems) and PD-1-AF488, Ly6G-PE, and CD3-APC (Biolegend). Images were captured with a Zeiss LSM 880 confocal microscope. Image processing and quantification of the fluorescent intensity was performed with ImageJ and ZEN 2.1 Lite software.

### RNA Extraction and RT-qPCR

Total RNA extraction from snap-frozen kidney and spleen tissues (pre-weighed), as well as enriched neutrophils, was performed as we previously reported (30). Briefly, samples were homogenized in Qiazol lysis reagent (Qiagen) using a bullet blender homogenizer (Next Advance, Troy, NY). Total RNA was extracted using RNeasy Plus Universal Kit (Qiagen) that also ensured gDNA elimination. All procedures were performed according to the manufacturer’s instructions. Reverse transcription (RT) was performed by using iScript™ Reverse Transcription Supermix (Bio-Rad). Quantitative PCR (qPCR) was performed with PowerUp™ SYBR^®^ Green Master mix and an ABI 7500 Fast Real-Time PCR System (Applied Biosystems). Relative expression of different transcripts was calculated using the 2−ΔΔCt method with 18S rRNA as the housekeeping gene. Data are shown as ΔCt values. Primer sequences for mouse IL-6, TNFα, IL-1β, BAFF, IL-6 receptor, gp-130 (IL-6 signaling transductor), PD-L1, CXCR4, and CXCR2 are shown in **Table S2**.

### ELISA

Anti-double-stranded (ds)DNA IgG levels in diluted serum samples was determined following a previously reported ELISA assay (36). Total serum IgG levels were determined with a commercial kit (Bethyl Laboratories). Serum TNFα and IL-6 levels were measured with Biolegend’s ELISA MAX kits.

### Statistical Analysis

Student’s *t* test was employed for the comparison between groups. Specifically, unpaired *t* test was used for the first experimental setting (see below for details); whereas paired *t* test was used for the second experimental setting since littermates were used to generate the two experimental groups. Asterisks were used to indicate significant differences (P < 0.05), and the unequal sign (≠) indicates nearly significant P-values (P < 0.10). All analyses were performed with Prism GraphPad.

## Results

### Reduced Lupus-Associated Splenomegaly With Ambient Exposure to QACs

**Figure 1:**
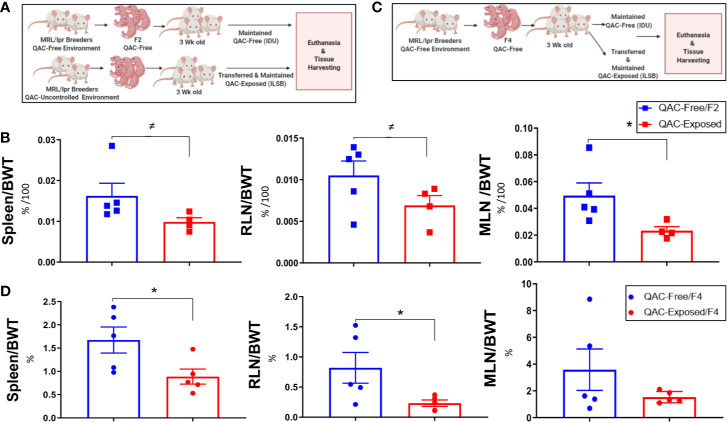
Reduced lupus-associated splenomegaly with ambient exposure to QACs. **(A)** Study design of the 1st experimental setting. See text for details. **(B)** Weight of lymphoid organs shown as ratios to the body weight at 16 weeks of age. RLN, renal lymph node. MLN, mesenteric lymph node. **(C)** Study design of the 2nd experimental setting. **(D)** Weight of lymphoid organs shown as ratios to the body weight at 12 weeks of age. Data are represented as mean ± SEM. ^≠^P < 0.10, *P < 0.05.

We had previously observed that QACs increased the production of pro-inflammatory cytokines from macrophages (21). Thus, we hypothesized that they would exacerbate autoimmunity and investigated whether ambient exposure to QACs in the form of a disinfectant would affect disease pathogenesis in lupus-prone MRL/lpr mice. We performed the study in two experimental settings to try to eliminate confounding variables generated from the fact that we had to house the QAC-free and QAC-exposed mice in different animal facilities. In the first experimental setting, we compared the F2 generation of QAC-free MRL/lpr with age-matched QAC-exposed mice (**Figure 1A**). These QAC-exposed mice were bred in a facility with minimal QAC use (Phase IV). Exposure was through the caging materials which picked up residual QACs through the cage wash. These mice received limited uncontrolled environmental exposure throughout breeding and gestation before being transferred at weaning to a full-use QAC facility (ILSB). The QAC levels of these facilities are shown in **Table S1**. Surprisingly, QAC-exposed mice had less severe splenomegaly and lymphadenopathy than QAC-free mice at the endpoint of 16 weeks of age (**Figure 1B**). We repeated this experiment and found similarly significant decreases in the sizes of the spleen and renal lymph node (RLN), as well as a nearly significant reduction of mesenteric lymph node (MLN) (P = 0.0528) in QAC-exposed mice at 14 weeks of age (**Figure S2**). In the second experimental setting, both groups of mice were born in the QAC-free facility as the 4th generation of QAC-free MRL/lpr mice. Some were transferred to the QAC-use facility (ILSB) at weaning that constituted the QAC-exposed group (**Figure 1C**). Similar results were observed; QAC-exposed mice had significantly smaller spleens and RLNs, and a trending decrease of MLN-to-body weight ratio at the endpoint of 12 weeks of age (**Figure 1D**). We also repeated the second experiment for a different endpoint of 16 weeks of age; the differences were not statistically significant, but the trend was consistent (data not shown). Therefore, contrary to our initial hypothesis, ambient exposure to QACs decreased two hallmarks of lupus, splenomegaly and lymphadenopathy, in female MRL/lpr mice. We decided to focus on the spleen for mechanistic investigations.

### Decreased *Ex Vivo* Migration and Chemotaxis of Neutrophils With Ambient Exposure to QACs

**Figure 2:**
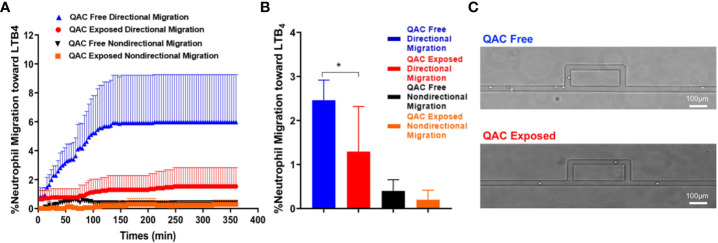
Decreased *ex-vivo* migration and chemotaxis of neutrophils with ambient exposure to QACs. **(A)** Pattern of the competitive chemotaxis toward LTB_4_ as determined by a microfluidic μC3 assay. **(B)** Percentage of migrated neutrophils toward LTB_4_. **(C)** Representative micrographs showing directional (straight channels) vs. non-directional migration (cells were lost within the maze). Data obtained from 12-week-old mice are shown as mean ± SEM. n = 5/group, *P < 0.05.

Neutrophils can shape SLE pathogenesis (37–39) and are one of the most abundant leukocytes in the bronchoalveolar lavage of inflamed airways following QAC exposure in mice (40). Neutrophils migrate and infiltrate lymphoid organs to modulate T-cell responses (41) that in turn facilitate SLE development (42). We compared the migratory capabilities of neutrophils isolated from the BM of QAC-exposed vs. QAC-free mice. We used a novel microfluidic chemotaxis platform (**Figure S1**), which allowed *ex-vivo* monitoring of the behavior of individual neutrophils while mimicking the mechanical confinement within animal tissues, and employed time-lapse imaging to measure the migration of BM-derived neutrophils. Competitive chemotaxis of neutrophils toward either the medium control or an inflammatory chemoattractant, LTB_4_, was determined. The results showed that neutrophils from QAC-exposed mice had significantly less directional migration toward LTB_4_ (**Figures 2A, B**). Notably, neutrophil from QAC-exposed mice exhibited a constantly low velocity of migration (**Figure 2A**), indicating that the maze on the microfluidic device might have hindered cell migration (**Figure 2C**); whereas neutrophils from QAC-free mice showed a sharp increase of velocity toward the LTB_4_ during the first 150 min. No significant differences in non-directional migration was observed. Similar results were obtained for neutrophils isolated at either 12 weeks (**Figure 2**) or 16 weeks of age (**Figure S3**). These results suggest that neutrophils from QAC-exposed mice have decreased capabilities for chemotaxic migration compared to those isolated from QAC-free mice.

### Decreased Infiltration of Neutrophils “Into the Spleen” With Ambient Exposure to QACs

**Figure 3:**
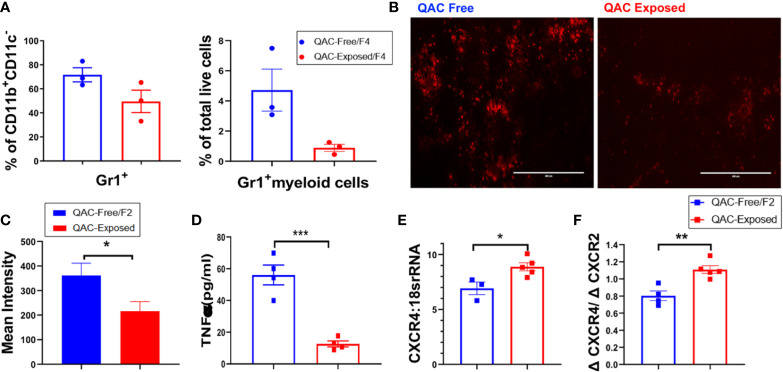
Decreased infiltration of neutrophils into the spleen with ambient exposure to QACs. **(A)** Splenic Gr-1+ cells as the percentage of CD11b+CD11c− cells (left) and Gr-1+ myeloid cells as the percentage of total leukocytes (right). **(B)** Representative micrographs of IHC-stained splenic sections showing Ly6G+ neutrophils. **(C)** Quantification of the micrographs shown as the mean intensity of PE fluorescence. **(D)** Serum level of TNFα. **(E)** Transcript level of CXCR4 in BM-enriched neutrophils. **(F)** Ratio of the transcript levels of CXCR4 and CXCR2. Data are represented as mean ± SEM. *P < 0.05, **P < 0.01, ***P < 0.001.

We hypothesized that with decreased migration and chemotaxis capabilities, fewer neutrophils would migrate from the BM to the spleen, leading to reduced splenomegaly. Therefore, we quantified neutrophils in splenic sections harvested from QAC-exposed vs. QAC-free MRL/lpr mice using flow cytometry. Both experimental settings gave us the same result. Using the 2^nd^ experimental setting as an example, when compared to their littermates that stayed in the QAC-free environment, we found a trending reduction of Gr-1+ cells as a percentage of CD11b+CD11c− myeloid cells or total live cells in the spleen of QAC-exposed mice (**Figure 3A**; gating strategy can be found in **Figure S6A**). IHC staining of splenic sections targeting Ly6G, a neutrophil-specific marker, confirmed that there were fewer neutrophils (**Figure 3B**), as indicated by significantly lower integrated density scores of the PE fluorescence (**Figure 3C**), in the spleens of QAC-exposed mice.

Cytokines modulate leukocyte migration to the lymphoid organs and subsequently shape SLE pathogenesis (43, 44). In particular, chemotactic factors such as TNFα can promote neutrophil migration and their tissue influx (45, 46), and prevent their egression from the BM (47). Consistent with decreased neutrophil infiltration into the spleen, serum TNFα were significantly reduced in QAC-exposed mice (**Figure 3D** and **Figure S4A**). To induce cell mobilization from the BM, TNFα inhibits the production of stromal cell-derived factor 1 (SDF-1; also called CXCL12) and downregulate the retention of CXCR4-expressing cells (48). Therefore, a lower serum level of TNFα suggests higher SDF-1 in the BM and decreased cell mobilization. Upregulation of CXCR4 also leads to cell retention in the BM (49, 50). CXCR2, on the other hand, antagonizes the effect of CXCR4 on cell trafficking (51, 52). Interestingly, we found that BM-derived neutrophils from QAC-exposed mice had a significantly increased transcript level of CXCR4 (**Figure 3E**) and a slight trending reduction of CXCR2 (**Figure S4B**), resulting in a significant increase of their ratio (**Figure 3F**) when compared to QAC-free animals. This suggests increased retention of neutrophils in the BM with ambient QAC exposure, which is consistent with the decrease in splenic infiltration.

Together, these findings suggest that in a QAC-exposed environment, there was decreased infiltration of neutrophils into the spleen.

### Pro-Inflammatory Neutrophilic Phenotype With Ambient Exposure to QACs

**Figure 4:**
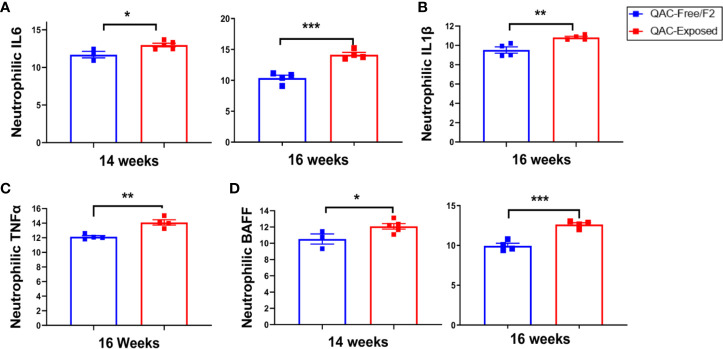
Pro-inflammatory neutrophilic phenotype with ambient exposure to QACs. Neutrophils were enriched from the bone marrow and relative transcript levels of **(A)** IL-6, **(B)** IL-1β, **(C)** TNFα, and **(D)** BAFF are shown. Data from the 1^st^ experimental setting are shown as mean ± SEM. *P < 0.05, **P < 0.01, ***P < 0.001.

Next, we questioned whether neutrophils from QAC-exposed MRL/lpr mice not only failed to migrate, but were also anti-inflammatory. Surprisingly, transcript analysis of BM-derived neutrophils showed that those from QAC-exposed mice had a significantly increased pro-inflammatory profile (**Figure 4**). Significant upregulation of IL-6 expression in isolated neutrophils was detected with QAC exposure (**Figure 4A**). Similar upregulation of neutrophilic IL-1β (**Figure 4B**) and TNFα (**Figure 4C**) was also observed. In addition, neutrophils from these mice had upregulated expression of BAFF (**Figure 4D**), a B-cell stimulator (53, 54), although no difference in the circulatory autoantibodies was observed (**Figure S4C**). This could be explained by the QAC exposure-associated reduction of the frequency of plasma cells (**Figure S4D**), which suggests that the extra BAFF was not effective in inducing antibody-secreting B cells. The frequency of plasmablasts was not changed (**Figure S4E**).

Concerned that the pro-inflammatory neutrophils in QAC-exposed mice would lead to kidney dysfunction associated with lupus (37, 55, 56), we investigated the severity of glomerulonephritis. The result showed that mice in the QAC-exposed vs. QAC-free environment had similar levels of proteinuria (**Figure S5A**). Interestingly, while neutrophils were producing more IL-6 (**Figure 4A**), renal expression of IL-6 receptor was significantly lower (**Figure S5B**) with ambient QAC exposure. A significant decrease was also observed for the IL-6 signaling transductor, gp130 (**Figure S5C**), in the kidneys of QAC-exposed mice. Renal inflammation activity and chronicity scores (**Figure S5D**), based on histopathological analysis of the kidneys (**Figure S5E**), did not significantly change with ambient QAC exposure.

These results suggest that while BM-derived neutrophils exhibit a pro-inflammatory phenotype in QAC-exposed mice, they did not exacerbate lupus-associated kidney inflammation.

### Reduced Expansion and Activation of Splenic T Cells With Ambient Exposure to QACs

**Figure 5:**
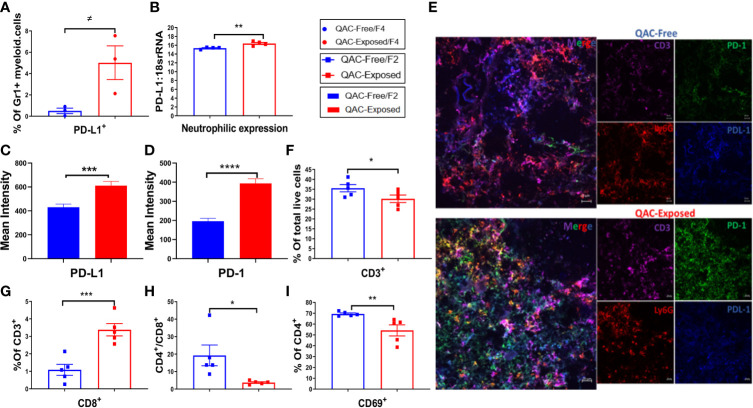
Reduced expansion and activation of splenic T cells with ambient exposure to QACs. **(A)** Percentage of splenic Gr-1+ myeloid cells expressing PD-L1. **(B)** Neutrophilic transcript level of PD-L1. (C–D) Quantification of the mean intensity of PD-L1 **(C)** and PD-1 **(D)** fluorescence in IHC-stained splenic sections. **(E)** Representative micrographs of IHC-stained splenic sections showing the co-localization of T-cell (CD3) expression of PD-1 and neutrophil (Ly6G) expression of PD-L1. **(F)** Percentage of CD3+ splenic T cells in total live cells. **(G)** Percentage of CD8+ cells in splenic T cells. **(H)** The ratio of CD4/CD8. **(I)** Percentage of CD4+ T cells expressing the activation marker CD69. Data are represented as mean ± SEM. ^≠^P < 0.10, *P < 0.05, **P < 0.01, ***P < 0.001, ****P < 0.0001.

Neutrophils can either mitigate or accelerate SLE pathogenesis (37, 38). Pro-inflammatory neutrophils exhibit part of their pathogenic roles in SLE by infiltrating the lymphoid organs to prime pathogenic T cells (41, 42). However, with SLE progression, neutrophils also upregulate their expression of PD-L1, a programmed death ligand (57). PD-L1 expressing neutrophils contribute to T-cell exhaustion and inhibit the proliferation of T cells (41, 58, 59). Interestingly, we found more Gr-1+ myeloid cells in the BM positive for PD-L1 with QAC exposure (**Figure 5A** and **Figure S6A**). This was consistent with significantly upregulated expression of PD-L1 mRNA in isolated Ly6G+ neutrophils (**Figure 5B**). Indeed, in IHC-stained splenic sections of QAC-exposed mice, we found prominent upregulation and interaction between PD-L1 and PD-1 (**Figure S6B**; quantifications in **Figures 5C, D**). Further, we show co-localization of PD-L1, PD-1, Ly6G (neutrophils), and CD3 (T cells) with QAC exposure (**Figure 5E**).

Analysis of T cells revealed significantly fewer CD3+ in the spleen of QAC-exposed mice (**Figure 5F**). Although there was only a trending reduction of CD4+ cells as the percentage of CD3+ T lymphocytes (**Figure S6C**, P = 0.052) with QAC exposure, the percentage of CD8+ cells increased significantly (**Figure 5G** and **Figure S6D**). This resulted in a significantly reduced CD4/CD8 ratio for QAC-exposed splenocytes (**Figure 5H**). CD8+ T cells are generally considered protective in lupus, as depletion of CD8+ T cells in murine lupus through antibody neutralization or genetic knockout is associated with aggravated autoimmune progression (60–62). Thus, a reduced CD4/CD8 ratio is consistent with decreased splenomegaly with ambient QAC exposure. Moreover, the activation marker CD69 was significantly reduced on CD4+ T cells (**Figure 5I**) and double-negative (DN) T cells (**Figure S6E**). Furthermore, a significant percentage of splenic CD3+ cells from QAC-exposed mice were CD62L+CD44− naïve T cells (**Figure S6F**), resulting in a significant increase of the ratio of naïve vs. effector T cells (**Figure S6G**).

Together, these results indicate reduced splenic T-cell expansion and activation in the ambient QAC-exposed environment that may have resulted from increased PD-L1:PD-1 interaction between neutrophils and T cells.

### Increased Apoptosis of T Cells With Ambient Exposure to QACs

**Figure 6:**
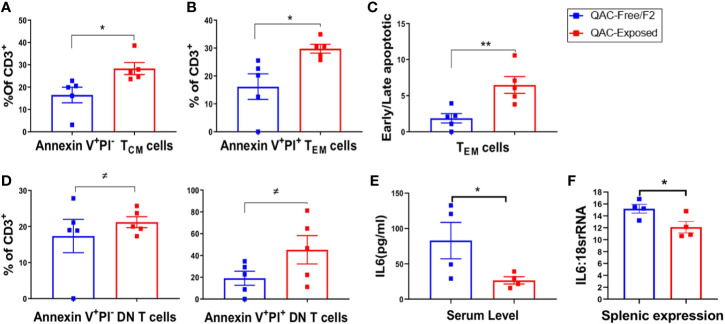
Increased apoptosis of T cells with ambient exposure to QACs. **(A)** Percentage of early apoptotic T_CM_ cells gated as AnnexinV+PI− on CD3+CD62L+CD44+ cells. **(B)** Percentage of late apoptotic or necrotic T_EM_ cells gated as AnnexinV+PI+ on CD3+CD62L-CD44+ cells. **(C)** Ratio of early apoptotic (AnnexinV+PI−) to late apoptotic or necrotic (AnnexinV+PI+) T_EM_ cells. **(D)** Percentage of early and late apoptotic DN-T cell as the percentage of CD3+ T lymphocytes. **(E)** Serum level of IL-6. **(F)** Transcript level of IL-6 in whole spleen. Data from the 1st experimental setting are shown as mean ± SEM. ^≠^P < 0.10, *P < 0.05, **P < 0.01.

QACs have been reported to significantly affect cell energetics and induce necrotic cell death (63). QAC-based cationic surfactants have been shown to induce apoptosis in both normal and cancer cells (64). In addition, increased PD-L1 expression on neutrophils may lead to T-cell exhaustion (58, 59). Therefore, we sought to determine increased apoptosis as an additional mechanism for the reduction of splenomegaly. Indeed, we found increased apoptosis of splenic memory T cells with ambient QAC exposure. Central memory CD62L+ CD44+ T_CM_ cells were at the early apoptotic stage as indicated by a significant increase of Annexin V+PI− cells (**Figure 6A**). Effector memory CD62L-CD44+ T_EM_ cells, on the other hand, were in the late apoptotic Annexin V+PI+ stage (**Figure 6B**). Additionally, there was a significant increase in the ratio of early to late apoptotic T_EM_ cells (**Figure 6C**). Similarly, we found a trending, nearly significant increase of both early apoptotic (P = 0.0549) and late apoptotic (P = 0.0547) DN T cells with QAC exposure (**Figure 6D**). Interestingly, there were significant reductions of serum (**Figure 6E**) and total splenic transcript (**Figure 6F**) levels of IL-6 with QAC exposure. IL-6 signaling can rescue T cells from apoptosis under both resting (65) and inflammatory (66) conditions. IL-6 also promotes T-cell survival through modulating the levels of anti-apoptotic factors (66, 67), and restricts activation-induced cell death of T cells *via* downregulating Fas/FasL signaling (68). Therefore, the reduction of serum and splenic IL-6 with QAC exposure is consistent with increased apoptosis of T cells.

To further evaluate the proliferation vs. apoptosis of splenic T cells, we stimulated them *ex vivo* using anti-CD3/anti-CD28. Although no difference in cell proliferation was noted after 3 days of stimulation (**Figure S7A**), significantly increased apoptosis of CD3+ T cells (**Figure S7B**) as well as CD4+ and CD8+ subpopulations (**Figures S7C–E**) were observed for splenocytes isolated from QAC-exposed mice. These results indicate increased apoptosis of splenic T cells with ambient QAC exposure, which might explain the decrease in splenomegaly.

## Discussion

The use of QACs has been correlated with the development of hypersensitivity responses (17, 18). This raises the concern that they may be related to autoimmune dysregulations. However, whether exposure to QACs perturbs immune homeostasis and contributes to development of autoimmune diseases based on the general concept of hygiene hypothesis remains speculative. To our knowledge, very limited studies have investigated the effect of QACs on systemic immune response (17, 20, 21), and our research was the first to explore how QAC-exposure would affect systemic autoimmunity. We evaluated the effects of the ambient exposure to QACs on lupus as a model of systemic autoimmunity. We chose the classical murine lupus-prone model, MRL/lpr, where mice develop splenomegaly, lymphadenopathy, and glomerulonephritis similar to the manifestations in human SLE (69–72). Although we cannot quantify ambient exposure as it varies from one day to another based on activities in the vivarium, it mimics real-life exposure. Unlike toxicological studies where animals are dosed with unrealistically high concentrations of QACs, assessing lupus progression following ambient exposure may allow the extrapolation of our results to SLE patients exposed to settings such as households and hospitals where QAC-based disinfectants are widely used (73). We monitored the progression of lupus in MRL/lpr mice that had been born and raised in completely QAC-free environment vs. QAC-exposed conditions. Our hypothesis was that exposure to QACs in the environment would deteriorate lupus pathogenesis through exacerbating neutrophilic responses. The rationale for this hypothesis was that neutrophils, one of the most abundant leukocytes in inflamed tissues following QAC exposure (40), play diverse pathological roles in lupus pathogenesis (37, 74–76). Surprisingly, however, we observed marked reduction of splenomegaly and lymphadenopathy with no significant changes on the progression of glomerulonephritis.

Neutrophils have heterogenic phenotypes and functions that may either accelerate or dampen the progression of lupus (37–39). To evaluate the roles of neutrophils in our model, we tested their migration *ex vivo*, as decreased migration and tissue infiltration of these cells could potentially contribute to decreases of splenomegaly and lymphadenopathy (43, 44). Indeed, we observed decreased directional migration of neutrophils isolated from QAC-exposed mice. Consistent with this, we found reduced infiltration of Ly6G+ cells in the spleens.

Paradoxically, BM-derived neutrophils from the exposed mice exhibited a pro-inflammatory phenotype. Upregulated neutrophilic expression of different modulators known to aggravate adaptive immune dysregulations in SLE (55, 77, 78) was observed including IL-6, IL-1β, TNFα, and BAFF. However, the upregulation of BAFF was not associated with increased systemic autoantibodies or kidney pathology. Instead, we observed a reduction of pathogenic T cells in SLE including the activated CD69+CD4+ T cells (79) and DN T cells (80, 81). Meanwhile, a significant increase of protective CD8+ T cells (60, 62) was observed. Moreover, there was a significant increase in the ratio of naïve to effector T cells. These data suggest that neutrophils infiltrating the spleen might have adopted suppressive functions and hindered T-cell activation and expansion that together contribute to reduced splenomegaly in QAC-exposed mice. This notion is supported by the upregulated expression of PD-L1 on the neutrophils with QAC exposure. PD-L1 expression on neutrophils has been shown to suppress T-cell responses in different inflammatory milieu such as in tumorigenic niches (82). Indeed, we detected prominent upregulation and co-localization of PD-L1 and PD-1 on neutrophils and T cells, respectively, with QAC exposure. In addition, PD-1:PD-L1 signaling is associated with not only reduced expansion of T cells (83), but also increased T-cell apoptosis (84). Interestingly, we also observed increased apoptosis of effector T-cell populations in the splenocytes of QAC-exposed mice upon *in-vitro* stimulation. This suggests that QAC-exposure may have hindered T-cell activation through neutrophilic PD-L1–induced signaling.

Importantly, apoptotic cells from QAC-exposed mice were found to be in the early apoptotic stages. We observed increased early apoptosis of splenic T_CM_ cells without significant changes in the late apoptotic T_CM_ cells. Early apoptosis is a signal for phagocytosis that does not provoke inflammatory responses (85). For apoptotic cells to provide signals for an autoimmune response, they would have to undergo late apoptotic stages in which cells lose their integrity allowing the leakage of cytosolic and nuclear contents that in turn activate immune cells (85, 86). Therefore, the pattern of apoptosis we observed in QAC-exposed mice (early > late) is consistent with less systemic inflammation as indicated by the reduction of serum IL-6 and TNFα.

Perturbation of immunological tolerance in SLE could be an outcome of many contributing factors, including defective apoptosis and/or increased proliferation of autoreactive immune cells (8–10, 69) and defective clearance of autoantigens or apoptotic products (22, 87). QACs have been previously reported to significantly affect cell energetics, inducing apoptosis and necrosis (22, 64). Therefore, the phenotype of reduced splenomegaly and lymphadenopathy, an indication of protection against a mouse model of SLE, with QAC exposure may be a result of neutrophil-mediated toning of T-cell proliferation and/or apoptosis. However, our findings also indicate that even ambient exposure to QACs could alter neutrophil and T-cell phenotypes, functions, and fate, raising concerns about the immunotoxicity of these chemicals.

## Data Availability Statement

The original contributions presented in the study are included in the article/**Supplementary Material**; further inquiries can be directed to the corresponding authors.

## Ethics Statement

The animal study was reviewed and approved by IACUC at Virginia Tech.

## Author Contributions

XL, CJ, and TH conceived the study. XL and LA designed the experiments. LA performed the experiments. XC-P, QM, BS, and PD contributed to mouse dissection and sample collection. MM performed the neutrophil *ex-vivo* microfluidic migration assay, whereas SL and ML quantified the neutrophil *ex-vivo* microfluidic migration. RS and LX performed the measurement of QAC residues. KE performed histopathological scoring. LA analyzed the data. LA, XL, CJ, and TH wrote the manuscript. All authors contributed to the article and approved the submitted version.

## Funding

Funding was received from National Institute of Arthritis and Musculoskeletal and Skin Diseases. We also would like to acknowledge the following funding sources. Environmental Pathology and Toxicology Training Grant funded by the National Institute of Environmental Health Sciences (T32-ES007032) for supporting RP. Seguin. The National Institute of General Medical Sciences of the National Institutes of Health under award number R35GM133610 for supporting M Moarefian.

## Conflict of Interest

The authors declare that the research was conducted in the absence of any commercial or financial relationships that could be construed as a potential conflict of interest.
