# Supplementary material for: Quaternary Ammonium Compound Disinfectants Reduce Lupus-Associated Splenomegaly by Targeting Neutrophil Migration and T-Cell Fate
Source: Front Immunol. 2020 Oct 21;11:575179. doi: 10.3389/fimmu.2020.575179 (PMC7609861; doi:10.3389/fimmu.2020.575179)
Supplement: Supplementary file 1 [file DataSheet_1.pdf]

Supplemental information

Table S1. The level of QAC residues in mouse cages.

			BAC (µg) per mouse box				
**Cages Type**	**Facility/Source**	**# of examined cages**	**C10-BAC**	**C12-BAC**	**C14-BAC**	**C16-BAC**	**DDAC**
**Disposable**	**Free**	11	0	0	0	0	0
**Polycarbonate**	**Uncontrolled (Carryover –cage washing)**	7	0	0.04	0.23	0.21	0.99
**Polycarbonate**	**Exposed (Ambient exposure)**	4	0	0.40	2.18	0.88	4.70

QAC residues including benzyl ammonium chloride (BAC) and didecyl dimethyl ammonium chloride (DDAC) were determined inside mouse cages using UPLC-MS/MS with deuterated internal standards after one week of use. The amounts of different residues were variable based on the facility (source of animal cages).

Table S2. Primer sequences

TARGET	Forward Sequence	Reverse Sequence
**IL-6**	TAGTCCTTCCTACCCCAATTCC	TTGGTCCTTAGGCACTCCTCC
**TNF α**	CCCTCACACTCAGATCATCTTCT	GCTACGACGTGGGCTACAG
**IL-1 β**	GCAACTGTTCCTGAACTCAACT	ATCTTTTGGGGTCCGTCAACT
**BAFF**	ACACTGCCCAACAATTCCTG	TCGTCTCCGTTGGGTGAAATC
**PD-L1**	GACCAGCTTTTGAAGGGAAAT	CTGGTTGATTTGCGGTATGG
**CXCR4**	TCAGTGGCTGACCTCCTCTT	CTTGGCCTTTGACTGTTGGT
**CXCR2**	GTGCATAGCCATGTGGTTAC	GGCAGGATACGCAGTACG
**IL6 Receptor**	CCTGTGTGGGGTTCCAGAGGAT	CTGCCAGTATTCTCAGCAGCTG
**IL6st(gp-130)**	GAGCTTCGAGCCATCCGGGC	AAGTTCGAGCCGCGCTGGAC
**18s rRNA**	GTAACCCGTTGAACCCCATT	CCATCCAATCGGTAGTAGCG

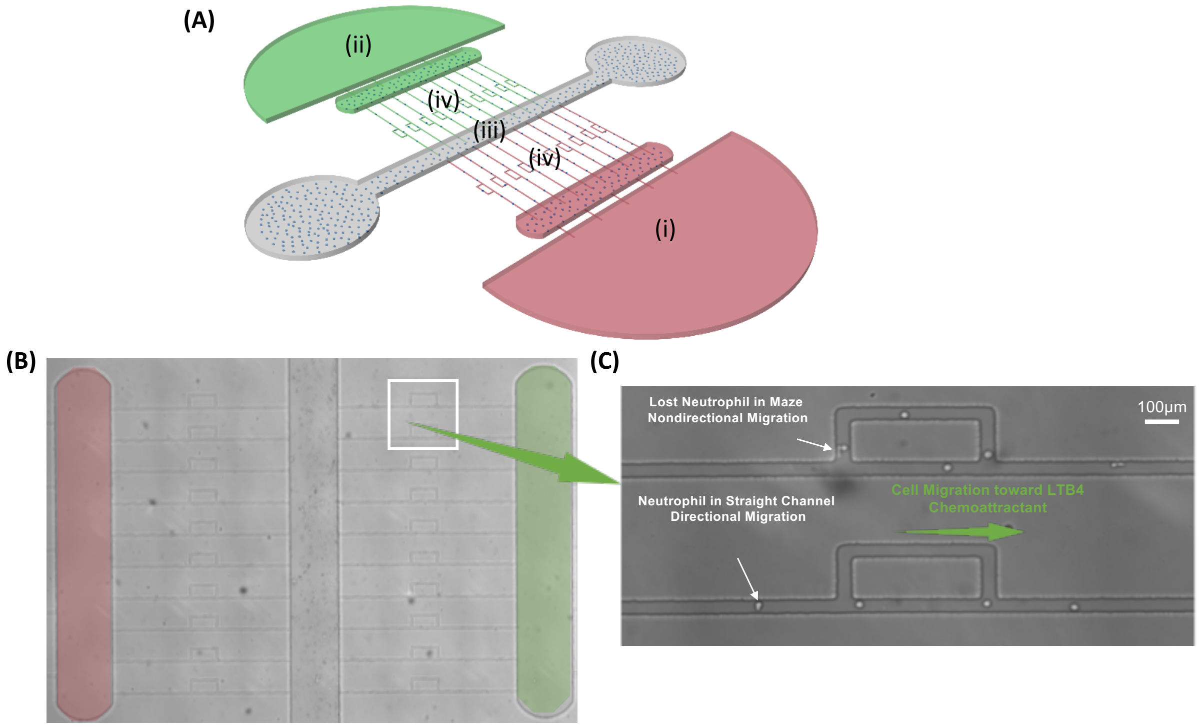

Figure S1. Microfluidic Competitive Chemotaxis-Chip (µC3). µC3 was designed with two chemoattractant reservoirs that enable the formation of dual, competitive chemoattractant gradients. (A) 3D schematic of the µC3 device setup allowing quantification of the percentage of migrating neutrophils and measurement of their directionality and migration velocity: (i) Control reservoir (medium) at the right end of the device; (ii) Chemoattractant reservoir at the opposite end; (iii) Central cell-loading channel for loading neutrophils; and (iv) Ten linear migration channels connecting the central cell-loading channel to both reservoirs. [Boribong, B.P., A. Rahimi, and C.N. Jones, Microfluidic Platform to Quantify Neutrophil Migratory Decision-Making. *Methods Mol Biol*, 2019. 1960: p. 113-122.] (B) Overlay microscopy image taken with Nikon TiE (10X). Chemoattractants were labeled with fluorescent dextran (FITC and TRITC) to allow visualization of the gradient formation. (C) Cell maze for measuring neutrophil directionality, and migration velocity and pattern. Cells that went through the linear channels migrated directionally, while those lost within the mazes were considered non-directionally migrating cells. Scale bar = 100 µm.

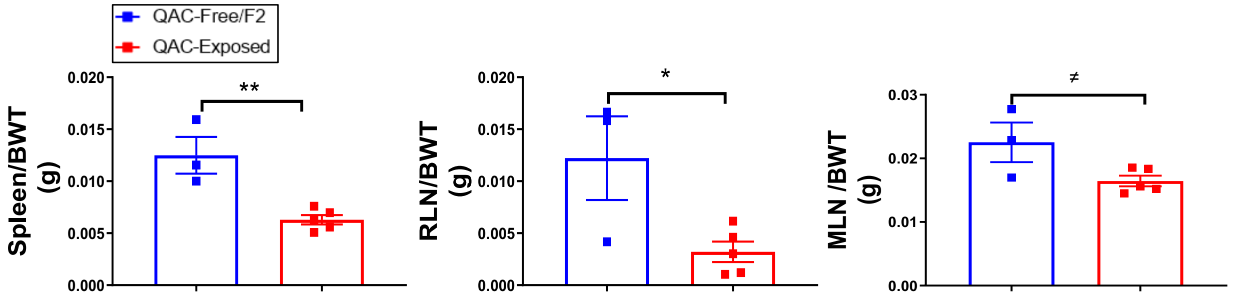

Figure S2. Splenic, renal lymph node (RLN) and mesenteric lymph node (MLN) weights relative to the body weight in the 1st experimental setting at 14 weeks of age. *P < 0.05, **P < 0.01, ≠P<0.10.

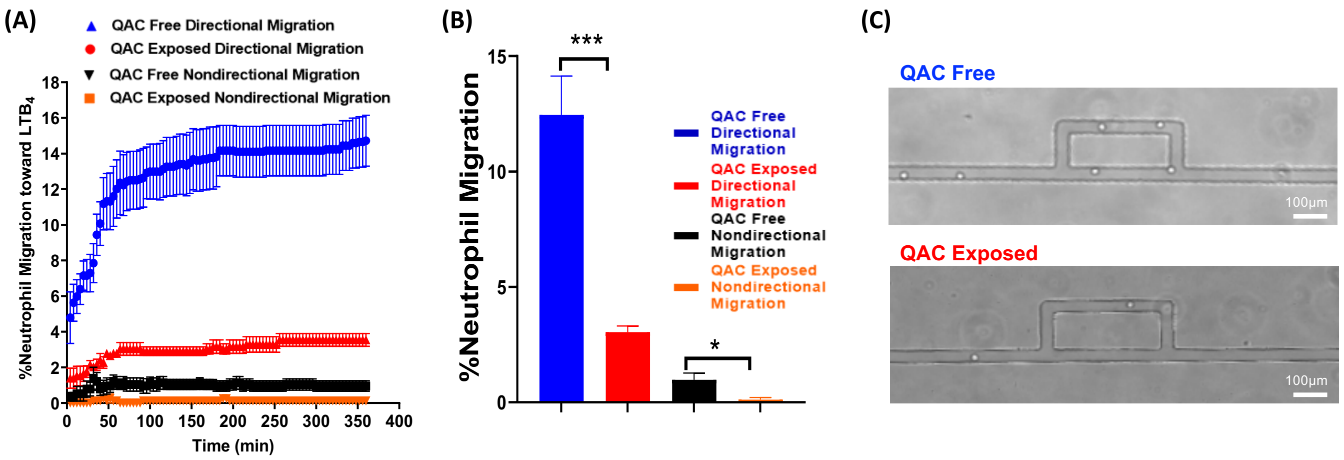

Figure S3. Chemotaxis of bone marrow-derived neutrophils isolated at 16 weeks of age. (A) Pattern of the competitive chemotaxis toward LTB_4 as determined by a microfluidic μC3 assay. (B) Percentage of migrated neutrophils toward LTB_4. (C) Representative micrographs showing directional (straight channels) *vs*. non-directional migration (cells were lost within the maze). n=5/group, $^*P<0.05$, $^{***}P<0.005$.

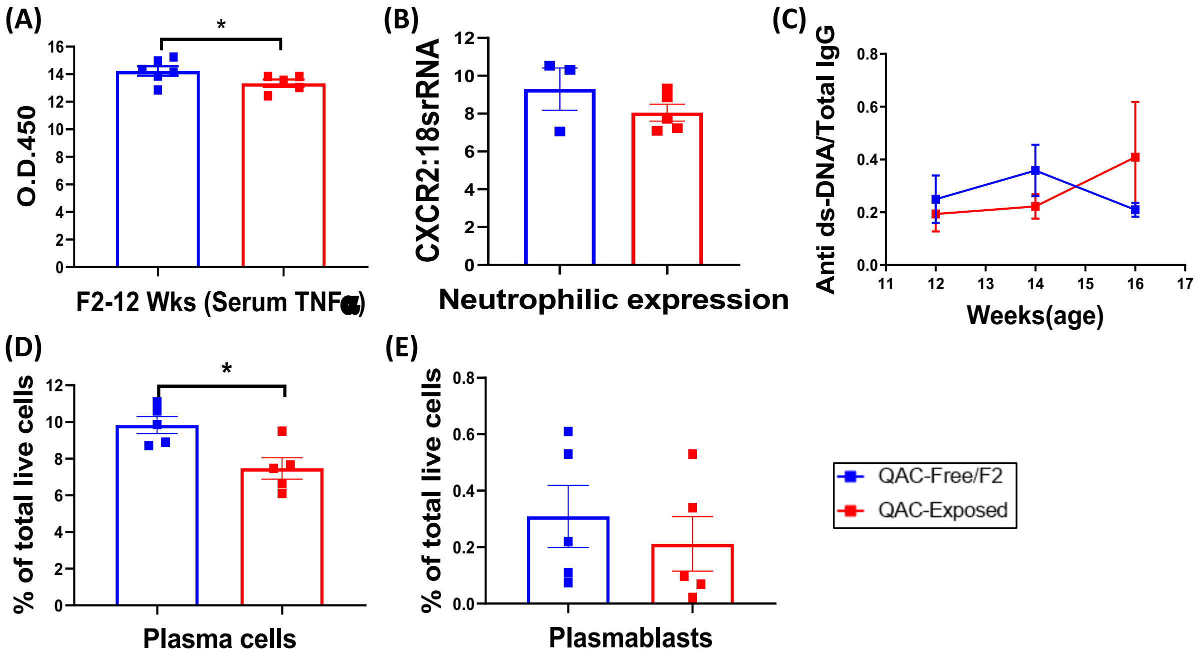

Figure S4. (A) Serum TNFα levels. (B) Transcript level of CXCR2 in bone marrow-enriched neutrophils as determined by RT-qPCR. (C) The ratio of anti-dsDNA IgG to total IgG. The percentage of (D) Plasma cells (gated as CD19-CD27-CD138+CD44+) and (E) Plasmablasts (gated as CD19+/*low*CD27+/*low*CD138+CD44+). Data are represented as mean ± SEM. $^*P < 0.05$.

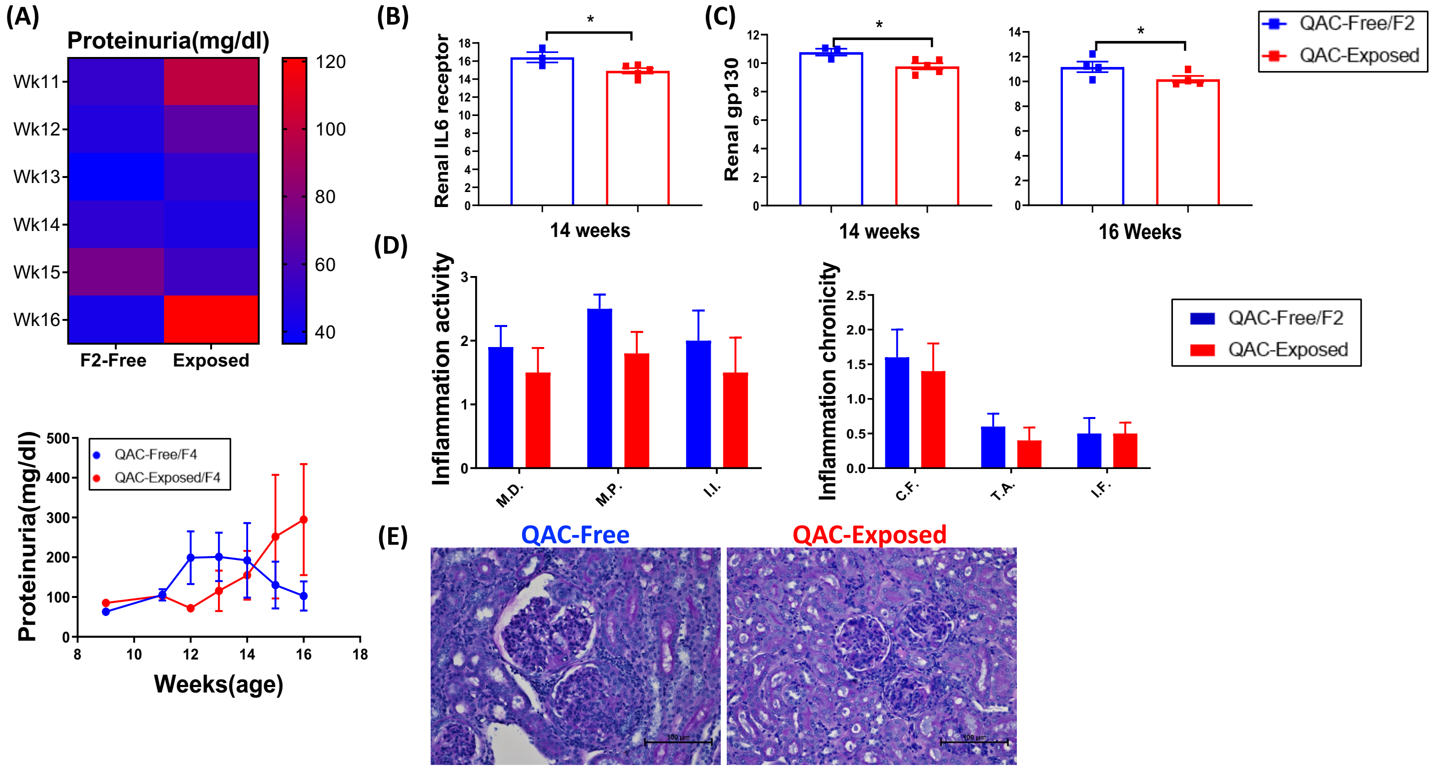

Figure S5. Assessment of glomerulonephritis. (A) Proteinuria levels in the 1st (upper) and 2nd (lower) experimental settings. (B) Transcript level of IL-6 receptor in the kidney. (C) Transcript level of IL-6 signaling transductor, gp130, in the kidney. (D) Renal histopathology evaluation shown as inflammation activity scores (M.D., mesangial deposits; M.P., mesangial proliferation; I.I., interstitial inflammation) and inflammation chronicity scores (C.F., crescent formation; T.A., tubular atrophy; I.F., interstitial fibrosis). (E) Representative micrographs of the kidney at 16 weeks of age. *$P<0.05$.

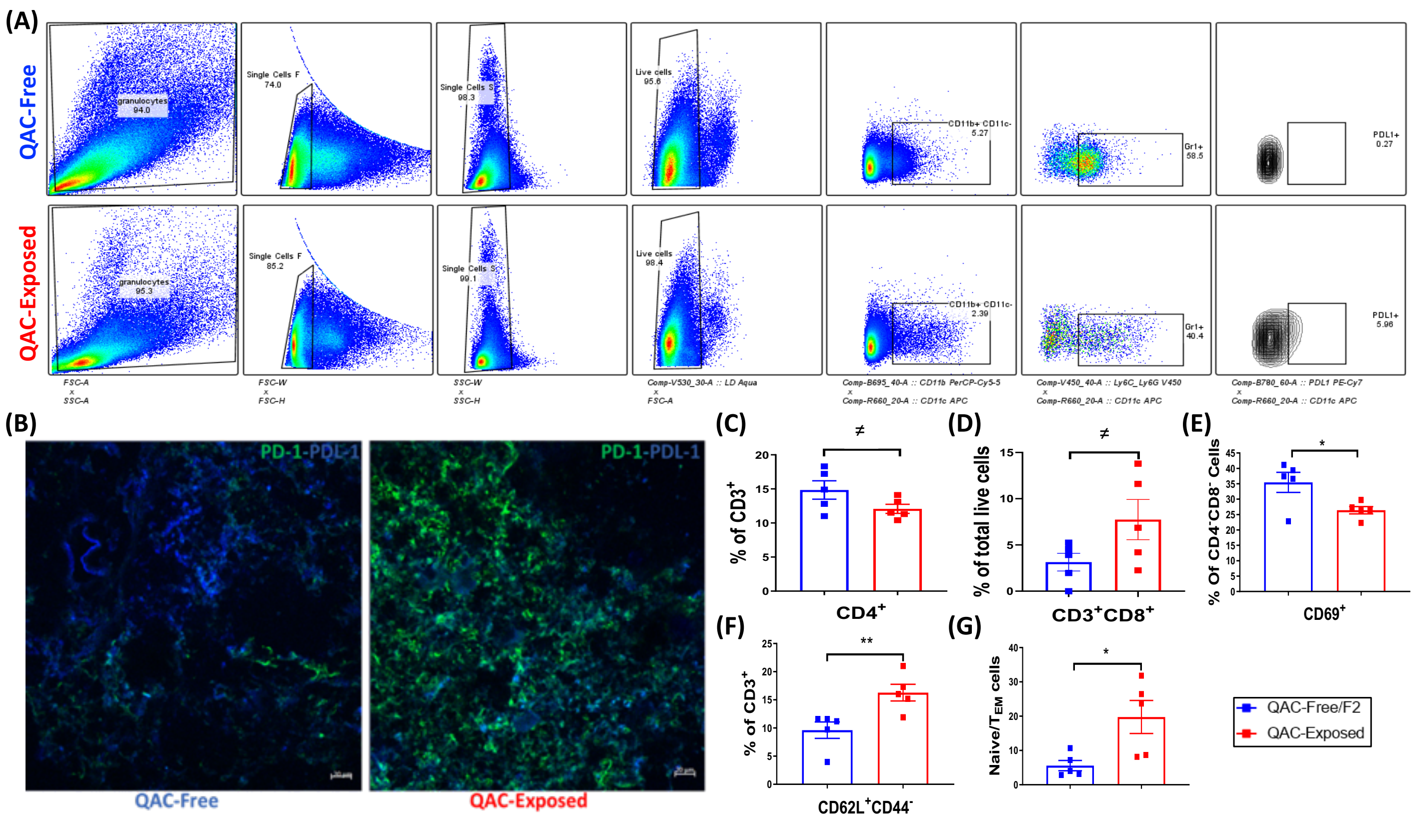

Figure S6. Analysis of T-cell expansion and activation. (A) Gating for PD-L1+ myeloid cells. (B) Representative micrographs of immunohistochemical stained splenic sections showing the colocalization of PD-1 (green) with PD-L1 (blue). Pictures were captured with a Zeiss LSM880 confocal microscope. (C-E) Splenocytes were isolated from 12-week-old MRL/lpr mice and assayed without stimulation. (C) Percentage of CD4+ cells. P = 0.0521. (D) Percentage of CD3+CD8+ lymphocytes from total live cells. (E) Percentage of DN-T cells expressing CD69. (F) Percentage of naïve CD62L+CD44- T cells. (G) The ratio of naïve to effector memory T cells (T_{EM}). Data are represented as mean ± SEM. $*P < 0.05$, $**P < 0.01$, $\neq P < 0.10$.

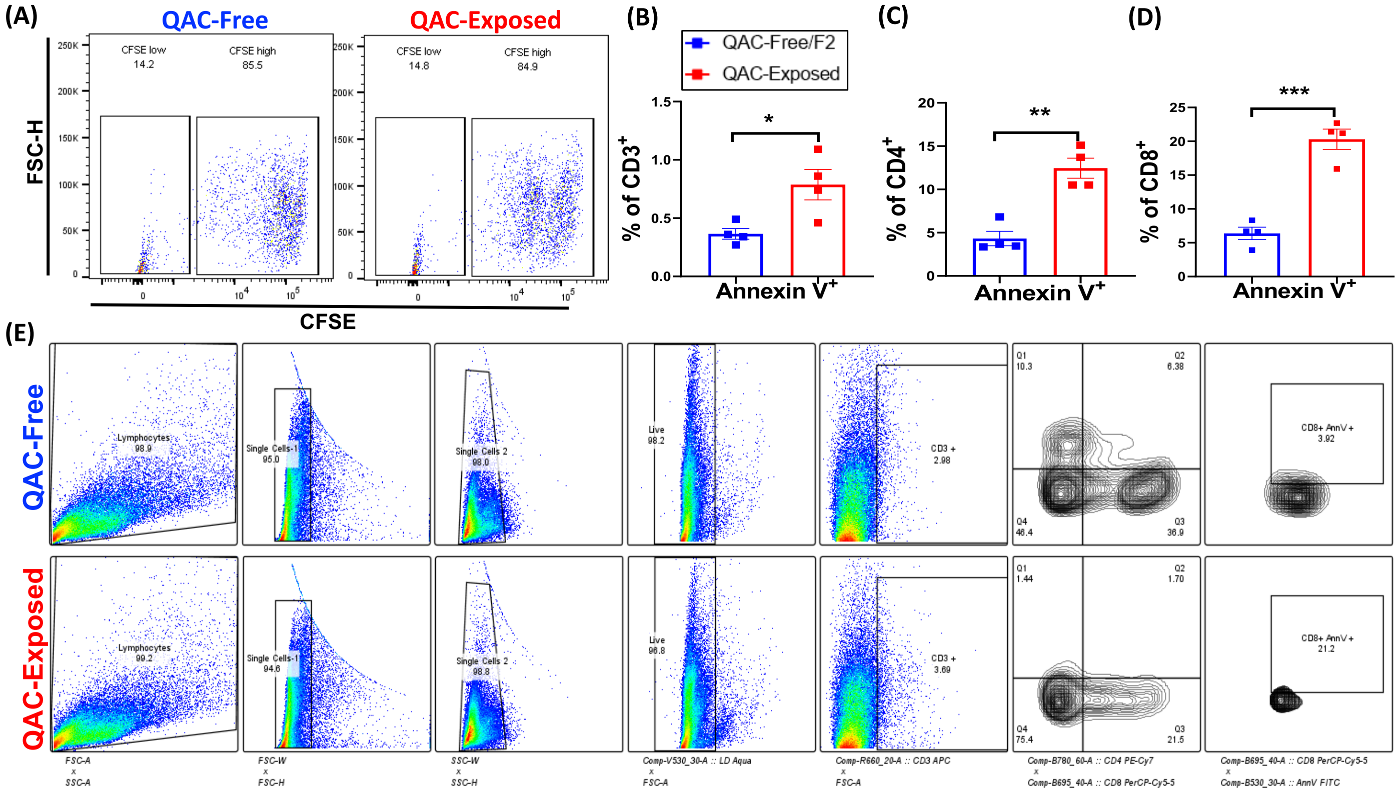

Figure S7. Analysis of T-cell fate. Splenocytes were isolated from 16-week-old MRL/lpr mice and stimulated with anti-CD3/anti-CD28 for 2-3 days. (A) Representative flow cytometry plot showing proliferation after 3 days of culture. (B-E) Annexin V+ cells as the percentage of CD3+ (B), CD4+ (C) and CD8+ (D) splenic T cells after 2 days of culture. (E) Gating strategy for Annexin V+CD8+ cells. Data are represented as mean ± SEM. *$P < 0.05$, **$P < 0.01$, ***$P < 0.001$.
